# Chemical exchange saturation transfer MRI shows low cerebral 2-deoxy-D-glucose uptake in a model of Alzheimer’s Disease

**DOI:** 10.1038/s41598-018-27839-7

**Published:** 2018-06-22

**Authors:** Daniele Tolomeo, Edoardo Micotti, Sonia Colombo Serra, Michael Chappell, Anniina Snellman, Gianluigi Forloni

**Affiliations:** 1 Laboratory of Biology of Neurodegenerative Disorders, Department of Neuroscience, IRCCS, Mario Negri Institute for Pharmacological Research, Milan, (MI) Italy; 2 Centro Ricerche Bracco, Bracco Imaging Spa, Colleretto Giacosa, (TO) Italy; 3 Department of Engineering Science, Institute of Biomedical Engineering, University of Oxford, 6396 Oxford, UK; 4 Medicity Research Laboratory, University of Turku, (Tykistökatu 6, FI-20510), Turku, Finland; 5 Turku PET Centre, University of Turku, (Kiinamyllynkatu 4-8, FI-20520,), Turku, Finland

## Abstract

Glucose is the central nervous system’s only energy source. Imaging techniques capable to detect pathological alterations of the brain metabolism are useful in different diagnostic processes. Such techniques are also beneficial for assessing the evaluation efficacy of therapies in pre-clinical and clinical stages of diseases. Chemical exchange saturation transfer (CEST) magnetic resonance imaging (MRI) is a possible alternative to positron emission tomography (PET) imaging that has been widely explored in cancer research in humans and animal models. We propose that pathological alterations in brain 2-deoxy-D-glucose (2DG) uptake, typical of neurodegenerative diseases, can be detected with CEST MRI. Transgenic mice overexpressing a mutated form of amyloid precusrsor protein (APP23), a model of Alzheimer’s disease, analyzed with CEST MRI showed a clear reduction of 2DG uptake in different brain regions. This was reminiscent of the cerebral condition observed in Alzheimer’s patients. The results indicate the feasibility of CEST for analyzing the brain metabolic state, with better image resolution than PET in experimental models.

## Introduction

Alzheimer’s disease (AD) is characterized by amyloid deposition in the brain parenchyma, intracellular fibrillary tangles formed by hyperphosphorylated tau and diffuse cerebral atrophy. In the diagnostic phase these features are identified by imaging analysis or cerebrospinal fluid (CSF) investigations, along with clinical evaluation of cognitive dysfunction by neuropsychological tests, to recognize AD with adequate accuracy^1^.

In the last decade the differential diagnosis between AD and other neurodegenerative disorders has been facilitated by the development of the positron emission tomography (PET) technique which is able to investigates brain metabolism by detecting the uptake of a radioactive glucose analog ([^18^F]2-fluoro-2-deoxy-D-glucose, [^18^F]FDG)^2–4^. The metabolic status measured by [^18^F]FDG-PET is combined with the use of different PET tracers (e.g. Pittsburgh compound B [^11^C]PIB, [^18^F]florbetapir or [^18^F]florbetaben) to identify the deposition of amyloid plaques. These have boosted the accuracy in the prediction of the conversion to AD in subjects with mild cognitive impairment (MCI)^2,5,6^. Regional analysis with PET have shown that in cortical areas [^11^C]PIB retention correlates inversely with glucose metabolism, measured with [^18^F]FDG^7,8^. More recently also several tau pathology imaging agents have been developed and investigated in clinical studies^9^.

In anticipation of large screening tests for risk assessment of the population at pre-clinical stages of the pathology, or long follow-up studies, PET imaging suffers certain drawbacks. It has low spatial resolution and needs special infrastructures to synthesize, distribute and dispose of radioactive tracers, making it expensive. Moreover, the ionizing radiation limits repeated measurements.

Magnetic Resonance Imaging (MRI) usually helps in AD diagnosis at later stage of the disease, when the brain shows the typical structural patterns of atrophy. Specific MRI techniques have now been developed that go beyond the measurement of brain shrinkage. Cerebral blood flow (CBF) and functional MRI (fMRI) techniques have been implemented to obtain functional biomarkers helping in early AD diagnosis^10,11^.

In translational approaches both PET and MRI biomarkers have been widely investigated in AD rodent models, validating both the animal models and the imaging techniques. The plaque burdens were longitudinally monitored^12–15^, with metabolic changes, by PET imaging. However, the PET images in rodent brains provide limited information because of the poor resolution. Furthermore, [^18^F]FDG-PET gave contradictory results in the AD animal models with no clear explanation^16–20^.

More consistent results have been obtained with the measure of MRI biomarkers: many transgenic mouse models have shown brain shrinkage during the progression of the disease^21–23^, along with changes in DTI parameters^24,25^ and alteration of brain metabolites measured with spectroscopy^26^. Recent papers have also shown modifications in functional biomarkers like CBF^15,27,28^ and resting state fMRI^29^.

An important step forward in exploring brain metabolism by MRI is chemical exchange saturation transfer (CEST) imaging of glucose. Recently, it has been shown that D-glucose can be used as an MRI contrast agent^30,31^. Results in cancer research with animal models and [^18^F]FDG-PET have been duplicated with CEST imaging, detecting the uptake in tumors of D-glucose^32^ and its homologous such as 2-deoxy-D-glucose (2DG)^33^, 3-O-methy-D-glucose (3OMG)^34^ and 2-amino-2-deoxy-D-glucose (GlcN)^35^.

The MRI signal source comes from water’s hydrogen nuclei excitation, since water is the most abundant source of hydrogen in the tissues. Information about other molecules can be obtained either directly by suppressing the water signal, like MR spectroscopy does, or indirectly with the CEST technique exploiting the natural equilibrium between water protons and those belonging to the surrounding molecules. Labile protons present in the sample are in fact able to exchange with water’s one contributing to the overall signal. Image contrast can be then modulated by applying a selective saturation pulse just before the acquisition. Reducing the contribution of other chemical species by saturating the sample at different frequencies, typically within 6 p.p.m. around the bulk water resonance, the so-called Z-spectrum can be measured and information about endogenous compounds obtained. This approach has been widely explored in the study of neurodegenerative diseases. Changes in glutamate and myo-inositol levels were detected in mouse models of AD^36–38^ and Huntington disease^39^. However, recent studies showed that the CEST signal drops rapidly after the injection of D-glucose, which it’s quickly metabolized by cells both in brain^40–42^ and tumor models^33^. Like [^18^F]FDG, 2DG is taken up in cells by the same transporters as glucose and phosporylated to 2-deoxy-D-glucose-6-phosphate (2DG6P), which remains trapped for many hours since it cannot be metabolized^43,44^.

Our aim was to show that pathological deficits in the brain metabolism of amyloid precursor protein (APP) transgenic mice can be highlighted by detecting differences in 2DG uptake. These changes can be observed with the CEST technique, carefully analyzing the Z-spectra using a multi-pool fitting procedure makes MRI a potential low cost alternative to PET imaging for studying neurodegenerative diseases.

## Results

**Figure 1:**
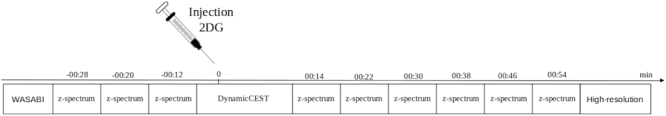
Timing of the experiment. (syringe image from https://www.dreamstime.com/stock-illustration-syringe-icon-white-background-image42045561, author: Konstantin Semenov).

The experimental conditions were settled up following the procedures previously described in tumor models investigations^33–35^. Fairly high concentrations of 2DG from 0.25 g/kg up to 2 g/kg were required to compensate the low sensitivity of the MRI technique. In preliminary experiments we tested 2DG doses in a range of 0.5–1 g/kg to optimize the analysis conditions. The higher dosage was not well tolerated in wild type (WT) mice since 2DG can lead to intracellular glucopenia obstructing glucose utilization and eventually to death. Subsequent experiments were done with a dose of 0.5 g/kg. All mice tested recovered in an hour after the experiments. Each experiment comprised two different paradigms (Fig. 1).

### Z-spectrum asymmetry changes

**Figure 2:**
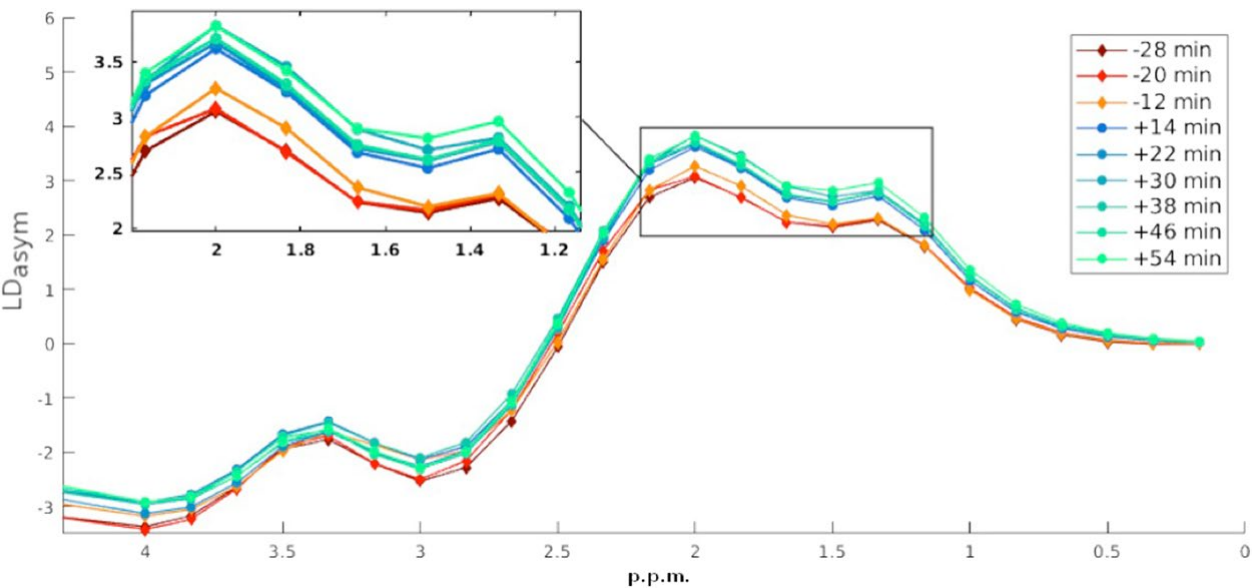
Asymmetry enhancement. Mean asymmetry fitted curves over the cortex region of a single WT mouse before and after 2DG injection. Highlighted by the square is the area where the cest enhancement has been evaluated.

We measured the accumulation of 2DG and therefore of 2DG6P in brain cells by examining the differences between three baseline Z-spectra acquired before the injection of the bolus and the six Z-spectra acquired after. Each one was fitted voxel-wise using the multi-pool fitting procedure described by the equation (3) and the lorentzian difference curve calculated using the equation (6). The glucose CEST enhancement (GCE) was than expressed as the change of the area under the lotentzian difference curve (AUC_LD_) between 2.3 and 1 p.p.m. that corresponds to the hydroxyl group resonating region (Fig. 2) and described by:

1 $$GCE(t)=[\frac{AU{C}_{LD}(t)-AU{C}_{LD}(Baseline)}{AU{C}_{LD}(Baseline)}]$$

**Figure 3:**
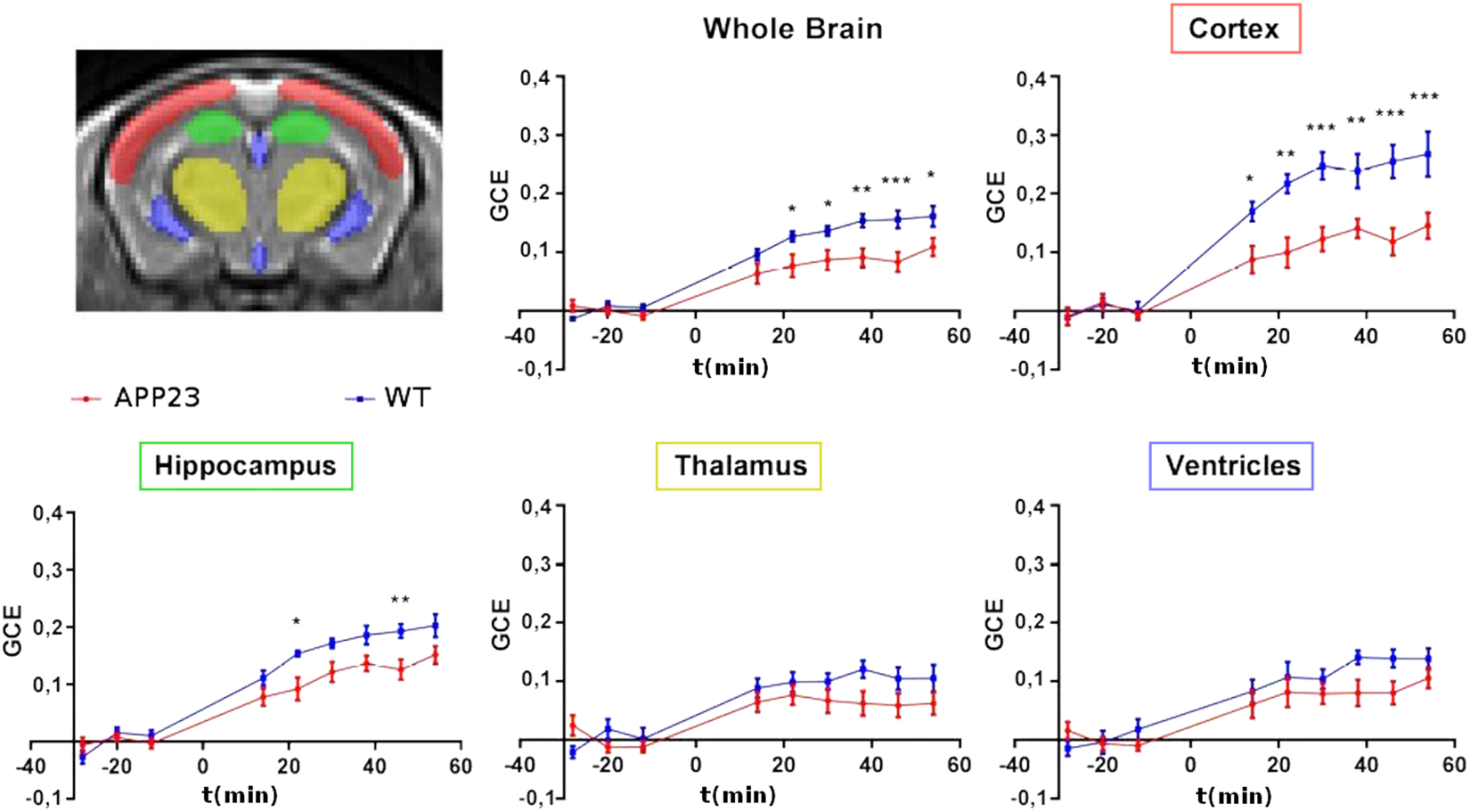
Temporal GCE. Group mean ± standard errors (s.e.m.) are visualized and injection time is indicated as zero. ***p < 0.001,**p < 0.01, *p < 0.05.

A custom template with an in-plane resolution of 0.16 × 0.16 mm^2^ was created by averaging all the unsaturated “high-resolution” slices acquired at the end of each experiment (using the buildtemplateparallel.sh script, provided by ANTs^45,46^). The unsaturated images of each Z-spectra were than co-registered with an affine transformation and resampled to this high-resolution template The transformations obtained were then applied to the images describing the GCE and the mean was calculated over selected regions of interest (ROI) traced over the template in the cortex, hippocampus, thalamus and ventricles (Fig. 3).

Two-way analysis of variance (ANOVA) followed by the Hochberg correction (R software, https://cran.r-project.org/) was done considering all time-points (Fig. 3). This enhancement reflects the uptake of 2DG. When the GCE was averaged all over the whole brain mask, statistical analysis indicated a significant effect of genotype (F = 35, p = 3.9 × 10^−8^), a significant effect of time (F = 41.44, p < 2.2 × 10^−16^) and a significant interaction genotype × time (F = 2.8, p = 0.007). Similarly, in the cortex, the analysis showed a significant effect of genotype (F = 56.46, p = 1.77 × 10^−11^), a significant effect of time (F = 36.1, p < 2.2 × 10^−16^) and a significant interaction genotype × time (F = 3.8, p = 0.0006). In the hippocampus time and genotype effects were also significantly different (respectively F = 61.5, p < 2.2 × 10^−16^ and F = 28.67, p = 4.9 × 10^−7^) as was the interaction genotype × time (F = 2.25, p = 0.03). In the thalamus and ventricles the single effects were significant but not the interaction genotype × time.

**Figure 4:**
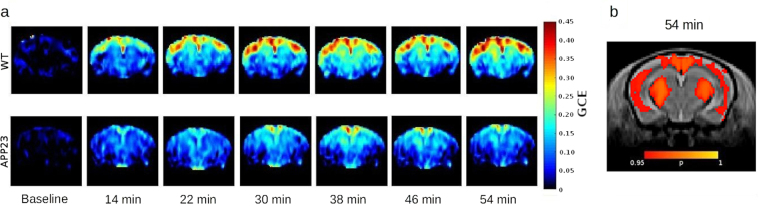
Graphic representation of the GCE (**a**) Time course represented as group mean images. Baseline is showed as the mean of the first three time points. (**b**) Voxel wise comparison. T-test on the normalized images representing the GCE 54 minutes after 2DG injection. In red the areas where WT > APP23 with p < 0.05.

Having verified that there was a significant difference between the two groups, we ran a voxel-wise student’s t test (Fig. 4b) on the co-registered images representing the GCE 54 minutes after injection using randomise^47^ embedded in the FSL software library^48^.

The cortex area of wild-type mice shows significantly greater CEST asymmetry than the APP23 transgenic mice (Fig. 4b). A similar difference was seen in the thalamus area, but does not appear in the ROI analysis where all the time points were considered.

A general reduction of CEST asymmetry in the brain of APP23 type mice has been observed with both approaches. Cortex is the region where there was the most significant effect.

### Continuous single offset acquisition

**Figure 5:**
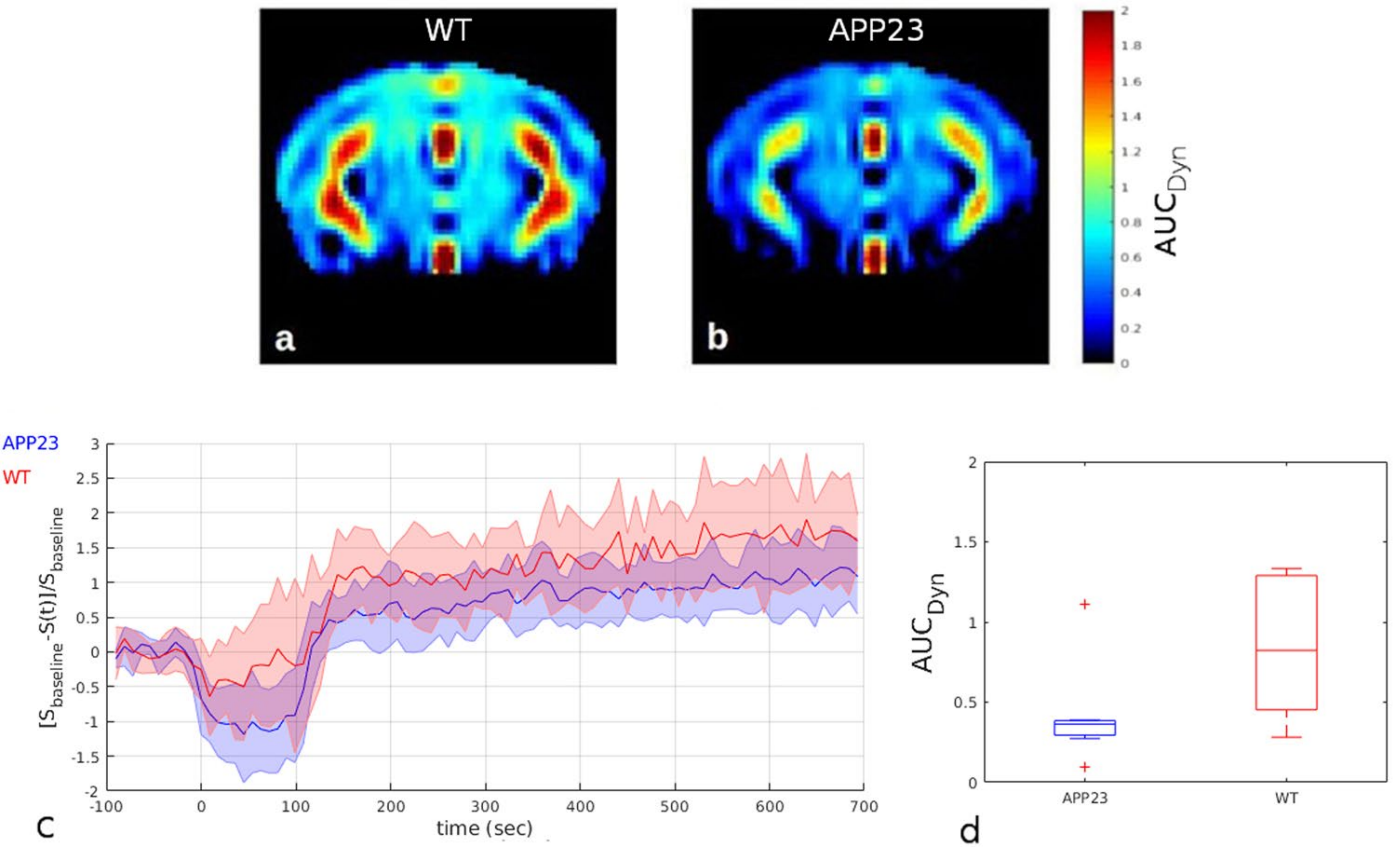
DynamicCEST measurements. (**a**,**b**) Group mean AUC_Dyn_ images normalized to the template. (**c**) group mean dynamic curves obtained by averaging the signal in the cortex. (**d**) AUC_Dyn_ calculated in the cortex area.

In order to reduce the sampling time and measure changes of the blood-brain volume dynamically a fast dynamic CEST acquisition was done following the indications of previous tumor studies in mouse models^49^ and humans^50^. Instead of measuring the whole Z-spectrum that takes 8 minutes each, we acquired 90 repetitions of the selected slice saturating just one frequency offset at 1.2 p.p.m. in the region of hydroxyl protons. One image was obtained every 9 seconds, which intensity depending to the amount of 2DG in the tissues. Injecting the bolus during the acquisition, the signal change (Fig. 5) can be established for each time post-injection taking account of the difference in the signal intensity from the baseline calculated averaging the first 15 images. The area under the curve (AUC_Dyn_) could be related to the amount of blood reaching the brain tissues. It was computed as the total sum of the differences of the signal from the baseline, starting from the end of the infusion (110 sec) following:

2 $$AU{C}_{Dyn}=\sum _{n={\rm{110}}}^{{\rm{700}}}[\frac{{S}_{baseline}-S({t}_{n})}{{S}_{baseline}}]$$

Images representing the AUC_Dyn_ were again co-registered to the template space and wild-type and transgenic mice compared using student’s t-test and the ROI approach. As showed in Fig. 5, a slight but not significant decrease of the AUC_Dyn_ in APP23 mice was detected, in the selected roi, after the 2DG infusion.

## Discussion

This study illustrates the feasibility of 2DG-CEST to detect impairment in brain metabolism in a PET-like investigation. Compared to previously published works on Alzheimer mouse models, we used 2DG, which remains internalized in the cells longer than glucose, enhancing the technique sensitivity. Recent papers performed on WT mice and rats^40–42^ demonstrated how 2DG-CEST could reach enough sensitivity to detect changes in brain glucose uptake in murine models. Our results indicates that CEST technique is able to highlight a reduction of 2DG brain uptake in 20-month-old APP23 mice compared to WT mice. Differences in the brain metabolic activity can be detected within a few minutes after the injection of 2DG, and persisted for over an hour (Fig. 3) in the cortex area. The blood 2DG contribution could be considered negligible and the increase in CEST asymmetry is mainly due to the extravascular concentration of 2DG and its metabolic product (2DG6P), which accumulates in cells.

This result, obtained in relatively old mice, opens the way to a longitudinal study in the same cohort of mice from juvenile to elderly stages to detect whether 2DG-CEST can be used as an early biomarker in the AD mouse model. The importance of the finding gains when related to the results in mice models with [^18^F]FDG-PET. In fact, according to the results in humans, hypometabolism is one of the earliest events in a subclinical stage of the pathology and progresses with age^7,51^. To our knowledge only one study has been done in APP23 mice with [^18^F]FDG-PET, showing no changes in glucose metabolism at 13 months of age^52^. Hypermetabolism was reported in 12-month-age APP/PS1^18^ and in 7-month-age Tg2576^53^ but it normalized with age. Hypometabolism was found in TASTPM^54–56^, APPPS1-21^20,57^ and 5xFAD^17^ mice. This variability may be linked to the intrinsic heterogeneity of transgenic mouse models that do not fully resemble the entire spectrum of AD pathological features. These animal models present different neuropathological features including cognitive impairment, neuroinflammation and plaque deposition in the brain and vessels but do not show neuronal cell death and intraneuronal fibrillary tangle deposition^58^. However, a sensitivity reduction might also be related to differences in microPET acquisition parameters and to environmental factors (e.g. temperature, fasting time, stress level) that could affect mouse metabolism adding variability between different studies^16,56,59^.

**Figure 6:**
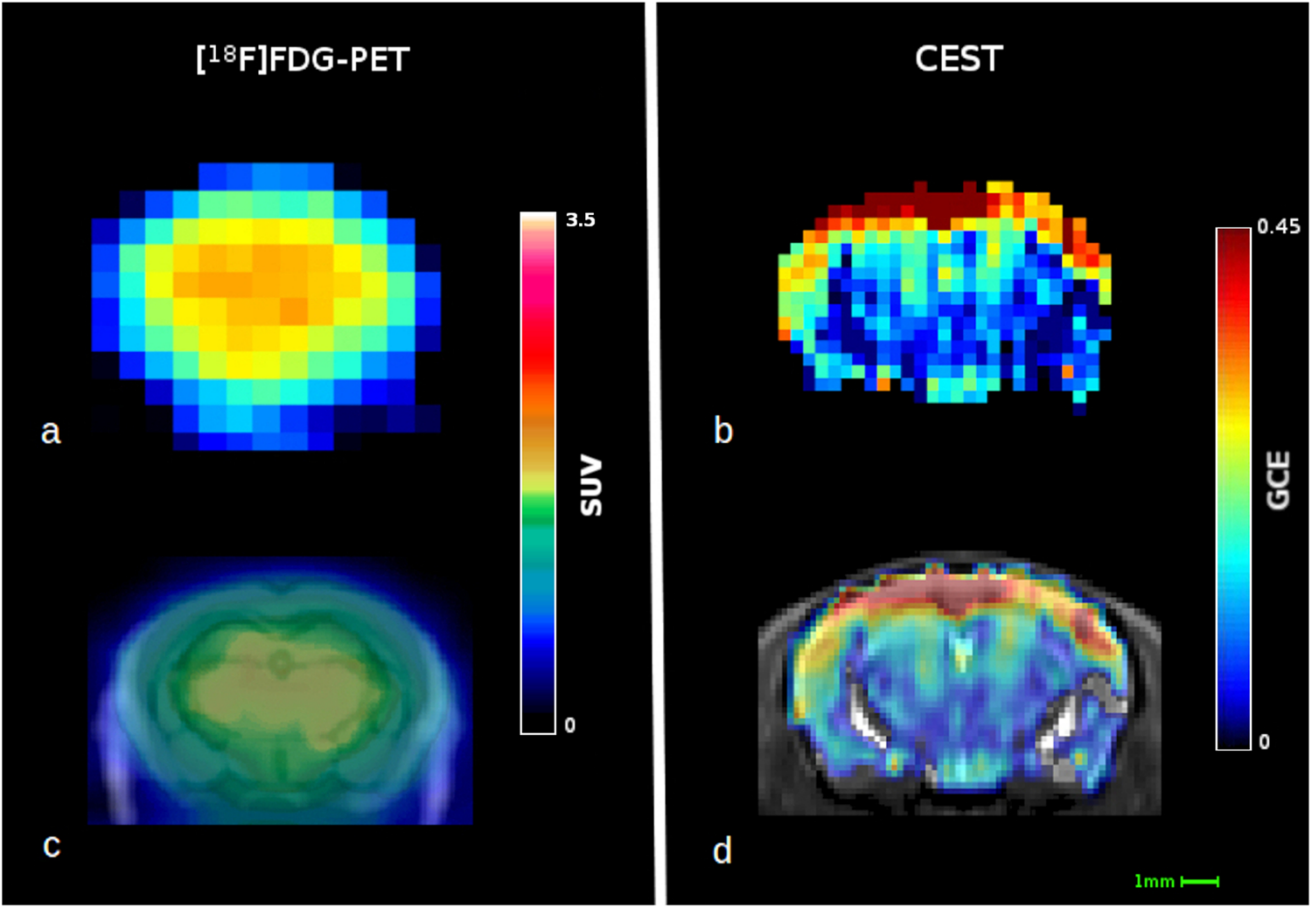
Fitting procedure. The left panel shows the location of the different compounds. Analysis firstly fitted the contribution of water and NOE (part A) and in a second step the other compounds contributions (part B).

Figure 6 shows a visual comparison between [^18^F]FDG-PET and CEST data. Even though, due to the methodological differences, it is difficult to directly compare the regional distribution observed with CEST to the regional [^18^F]FDG uptake obtained with PET in mice. Both techniques show intrinsic variability but PET can reach a modest resolution^60^ of 0.8 × 0.8 mm^2^ that makes a reliable differentiation between small regions of the mouse brain challenging. On the other side, with CEST, we have acquired images with an in-plane resolution of 0.35 × 0.35 mm^2^ but this could be further optimized just by extending the acquisition time or using coils with higher signal to noise ratio. In addition we cannot exclude that the different detected glucose distribution between the two techniques could be due to the rather high doses of 2DG needed by CEST, whereas PET utilizes non-pharmacological tracer doses. However, the pattern of accumulation observed with CEST could be compared more in detail with those given by [^14^C]-2DG or [^18^F]FDG autoradiography^57,61^ or those obtained by [^18^F]FDG-PET in rats, whose larger brains allows the easier differentiation of accumulation patterns in different brain regions^62^.

Our pipeline permits an automatic and robust data analysis. The correction of B_0_ and B_1_ inhomogeneities (done here using a WASABI^63^ sequence) is essential in order to translate the technique to humans, where static and excitation fields may present wide variability over the whole brain. The co-registration of GCE maps on a brain template enable to avoid mistakes due to manual tracing and permits a voxel-wise analysis.

Besides the steady-state acquisition, we have also acquired a dynamic acquisition covering the first 15 minutes after 2DG infusion (Fig. 5). We calculated the AUC_Dyn_ and no significant differences were detected in the dynamic acquisition during the infusion but there seemed to be a clear trend toward a more pronounced delivery of glucose in wild-type mice than transgenic mice. The interpretation of dynamic data is less straightforward than data obtained at longer times. To reduce the sampling time and observe dynamic changes in the blood flow, it is not possible to acquire the whole Z-spectrum and integrate it. The acquisition of a single saturation frequency repeated every 9 seconds is hindered by the impossibility of B_0_ and B_1_ corrections, making this kind of measurement much more sensitive to fluctuations, leading to less robust results. Analyzing the Z-spectrum with a multi-pool fitting procedure permits the calculation of the asymmetry curve over a region of interest in the frequency offsets, reducing the noise contribution in the evaluation of the effects observed. The signal change in the proximity of the injection may be the result of a mixed concentration of 2DG between extracellular glucose and the plasma fraction still present. Similarly ventricles shows an increased signal detected immediately after the injection through the dynamic acquisition (Fig. 5a,b). This effect disappears by the time of the first Z-spectrum acquisition (14 min after injection) which might be explained by a washout of 2DG from the ventricles where no accumulation is expected. Moreover, the slight though not significant reduction of AUC_Dyn_ detected in APP23 mice could be driven by a reduction of CBF in transgenic mice already described in APP23 mice in a late stage of the pathology^15^ indicating that this is not the best method to measure it within the context of the neurodegenerative diseases. Previous studies describing dynamicCEST acquisitions were made in tumor lesions where the massive blood volume and glucose consumption compared to healthy brain makes this technique feasible.

There are, however, few limitations in the reported study. Firstly, anesthesia is needed in preclinical studies, but it can inhibit glucose uptake in the brain^61,64^; the use of a mechanical ventilator could help to control physiological parameters and reduce the variability. Secondly, adopting slightly different saturation procedures, like the spin lock^65^, can enhance the sensitivity of this technique and so lower 2DG dose can be used. A similar approach has already been tested in oncology in humans^66,67^. In spite of these limitations 2DG-CEST offers a promising alternative [^18^F]FDG-PET for the study of neurodegenerative diseases. This work will be replicated in longitudinal studies and further experiments are needed to explore whether 2DG-CEST can be considered a powerful tool in drug discovery research, able to detect the changes in glucose metabolism induced by pharmacological treatments.

## Methods

### Animal preparation

All the procedures involving animals and their care were conducted according to European Union (EEC Council Directive 86/609, OJ L 358,1; December 12, 1987) and Italian (D.L. n.116, G.U., Suppl. 40, February 18, 1992) laws and policies, and in accordance with the United States Department of Agriculture Animal Welfare Act and the National Institutes of Health (Bethesda, MA, USA) policy on Humane Care and Use of Laboratory Animals. They were reviewed and approved by the Mario Negri Institute Animal Care and Use Committee that includes ad hoc members for ethical issues, and the Italian Ministry of Health. Mice were housed in standard conditions on a 12-hour dark/light cycle and fasted overnight, with free access to water before imaging. Experiments were carried out on 20-months-old APP23 mice (N = 7) over-expressing the human full-length AβPP (AβPP751) harboring the “Swedish” double mutation (K670N/M671L) and wild-type (N = 7) litter-mates (all bred on C57BL/6N genetic background)^58,68,69^. Respiration was monitored during the experiment and body temperature maintained at approximately 37 °C by a warm water circulating heating cradle. Mice were anesthetized with isoflurane (1.5%) in oxygen, the tail vein was cannulated with a catheter and connected to a 10% solution of 2DG (Santa Cruz Biotecnology). All mice fully recovered a few minutes after the end of the anesthesia. The animals were randomized and scanned over a period of eight days.

### MRI Data acquisition

MRI images were acquired on a 7T small-bore animal scanner (Bruker Biospec, Ettlingen, Germany) running ParaVision 5.1 and equipped with two actively decoupled radio frequency coils. A 7.2 cm diameter volume coil was used as the transmitter and a quadrature single channel surface coil as the receiver.

Before CEST measurements, pilot T_2_-weighted images were acquired for use as reference scan. A B_0_ field map was acquired and 1st and 2nd order shims adjusted using the MAPSHIM routine, over a voxel (5 × 7 × 7.5 mm^3^) set to cover the brain, excluding the olfactory bulb and cerebellum.

An unsaturated image was then acquired using a rapid acquisition with relaxation enhancement (RARE) sequence (TR/TE 5000/4.3 ms, RARE factor 24, single slice thickness 2 mm, matrix size 45 × 45, field of view 16 × 16 mm^2^, resulting in an in-plane resolution of 0.35 × 0.35 mm^2^). The same readout and geometry was used for subsequent scans. B_0_ and B_1_ inhomogeneities were measured using WASABI sequence^63^ with a continuous wave saturation pulse (B_1_ = 3,7 μT, t_sat_ = 5 ms, 43 frequency offsets between ± 1.5 p.p.m.).

CEST Z-spectra were measured over 58 frequency steps (300 ± 20 ± 5 ± 4.66 ± 4.33 ± 4 ± 3.83 ± 3.67 ± 3.5 ± 3.33 ± 3.17 ± 3 ± 2.83 ± 2.67 ± 2.5 ± 2.33 ± 2.17 ± 2 ± 1.83 ± 1.67 ± 1.5 ± 1.33 ± 1.167 ± 1 ± 0.83 ± 0.67 ± 0.5 ± 0.33 ± 0.167 0 p.p.m.) using a continuous wave saturation pulse (B_1_ = 1.5 μT, t_sat_ = 4 s) resulting in a total scan time per Z-spectrum of around 8 min.

The percentage signal change during the injection (Dynamic-CEST)^49,50^ was achieved by repeating the saturation (t_sat_ = 4 s, B_1_ = 1.5 μT) in the region of interest of the hydroxyl protons, 1.2 p.p.m. A bolus of 2DG solution (0.5 g/kg) was injected, without stopping the acquisition, after 15 baseline scans and over a period of 90 seconds. The dynamic acquisition covered a total of 90 repetitions and takes 13.5 minutes.

One last “high-resolution” unsaturated image was acquire with a RARE sequence (TR/TE = 5000/4.3 ms, RARE factor 16, slice thickness 2 mm, matrix size 90 × 90, field of view 16 × 16 mm^2^ resulting in an in-plane resolution of 0.18 × 0.18 mm^2^). Each experiment, from the induction of anesthesia, takes around 2 hours.

### MRI Data analysis

Analysis was done with MATLAB custom scripts to fit the signal voxel-wise within a mask brain region that was manually drawn using ITK-SNAP software^70^. B_0_ and B_1_ maps were calculated using the WASABI mode^63^. Each CEST Z-spectrum signal, expressed as a function of the frequency offset (Δω), was modeled as a sum of five inverted Lorentzian curves with amplitude (A_i_) and full width half maximum (L_i_).

3 $$Z({\rm{\Delta }}\omega )={\rm{1}}-\sum _{{\rm{1}}}^{{\rm{5}}}{A}_{i}{({\rm{1}}+{(\frac{{\rm{\Delta }}\omega -\delta {\omega }_{\iota }}{{\rm{0.5}}\cdot {L}_{i}})}^{{\rm{2}}})}^{-{\rm{1}}}+MT$$

This represents the effect of the saturation on the signal intensity directly on the free water molecules and from the exchanging amine (-NH_2_), amide (-NH), hydroxyl (-OH) and the signal of aliphatic protons (relayed nuclear Overhauser effect rNOE). While the semi-solid magnetization transfer contribution was considered just as an additional parameter since the saturation power is weak and calculated as:

4 $$MT=\frac{[{S}_{0}-{S}_{5p.p.m.}]}{{S}_{0}}$$

where S_0_ is the signal with saturation pulse at 300 p.p.m.

**Figure 7:**
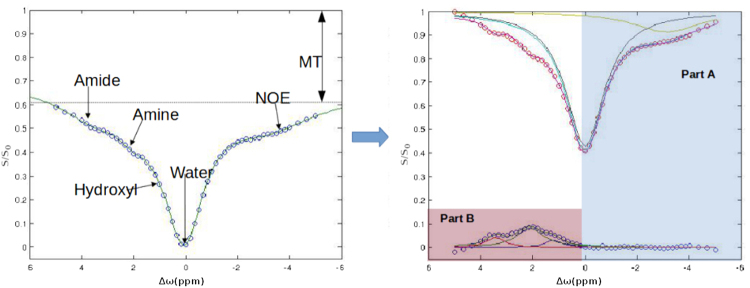
Visual comparison of the two techniques. (**a**) Single WT mouse PET imagerepresenting ^18^F-FDG uptake 50–60 minutes after injection with a voxel size of 0.78 × 0.78 × 0.8 mm^3^; (**b**) image representing the GCE of a single mouse 1 hour after the 2DG injection, it has a voxel size of 0.35 × 0.35 × 2 mm^3^; (**c**) PET image coregistered with computed tomography image and superimposed on a general mouse brain MRI template; (**d**) GCE image coregistered and superimposed on the in-house template. SUV = standard uptake value; GCE = glucose cest enhancement.

To ensure a reliable fit careful correction of the B_0_ inhomogenities the fit function (1) of the Z-spectrum signal was evaluated with a slightly different procedure from the previously published^71–75^ by splitting it into two subsequent steps as shown in Fig. 7. Direct water saturation and the contribution of NOE protons were firstly evaluated as the sum of two Lorentzian curves over the non-hydroxyl containing region using a subset of frequency offsets^76^ between -6 p.p.m. and 0.5 p.p.m. The water shift (δω) starting point was set using the previously calculated B_0_ map and signal drifts were corrected using the minimum of the water Lorentzian curve. Then the residue signal was fitted by the sum of the three remaining proton groups.

Since the asymmetry curve is defined as:

5 $$MT{R}_{asym}=\frac{{S}_{ref}-{S}_{lab}}{{S}_{{\rm{0}}}}$$

where S_ref_ and S_lab_ are the signal intensity and the label intensity of the acquired Z-spectrum and S_0_ is the unsaturated signal intensity, a Lorentzian Difference (LD)^77^ was computed as:

6 $$LD={Z}_{ref}-{Z}_{lab}$$

where Z_ref_ and Z_lab_ are the corresponding images with intensities given by the fit. In addition a linear correction for B_1_ was applied by multiplying the asymmetry curve by the previously relative B_1_ calculated. This simplified B_1_ correction was applied because we found small B_1_ differences.

### PET acquisition and processing

To make the visual comparison, shown in Fig. 6, between the resolutions of CEST and [^18^F]FDG-PET, one WT female mouse (26.7 g, age 12 months) was imaged with [^18^F]FDG using Inveon Multimodality PET/CT scanner (Siemens Medical Solutions, Knoxville, TN, USA). The mouse was fasted for 3 hours, anesthetized with 2.5% isoflurane and cannulated, before dynamic 60 minute PET scan in 3D-list mode was initiated at the time of intravenous [^18^F]FDG injection (6.3 MBq). Images were reconstructed using a 2-dimensional Filtered Back Projection algorithm and divided into 30 × 10 s; 15 × 60 s; 4 × 300 s and 2 × 600 s time frames. Obtained voxel size was 0.8 × 0.8 × 0.8 mm^3^. For the image used in Fig. 6a,c, time frame accounting for 50–60 minutes post injection was used, and in 6c, overlaid to a CT image of the same individual mouse, and a general mouse brain MRI template^78^.

The datasets generated and analyzed during the current study are available from the corresponding author on reasonable request.
